# A comparative analysis on risk of pulmonary hypertension in children with Atrio-ventricular (AV) canal defect: a multi-centre study

**DOI:** 10.4314/ahs.v22i1.28

**Published:** 2022-03

**Authors:** Josephat Maduabuchi Chinawa, Chika Onyinyechi Duru, Awoere Tamunosiki Chinawa, Bartholomew Friday Chukwu

**Affiliations:** 1 College of Medicine, Department of pediatrics, University of Nigeria/ University of Nigeria Teaching Hospital (UNTH), Ituku- Ozalla, Enugu State, Nigeria; 2 Senior Lecturer, Department of Paediatrics and Child health; 3 Niger Delta University Teaching Hospital Okolobiri Bayelsa State; 4 Enugu state University Teaching hospital, Enugu State

**Keywords:** AV canal defect, pulmonary hypertension, children, odds

## Abstract

**Objectives:**

This study is aimed at determining the risk of pulmonary hypertension in children with AV canal defect when compared with children with other congenital heart disease.

**Methods:**

A descriptive study carried out in three institutions over a six-year period among children who presented with AV canal defect and their controls who presented with other congenital heart defects.

**Results:**

A large proportion of the children with AV canal (77.5%) had pulmonary hypertension. Among the patients with pulmonary hypertension, 45.2% were males compared 54.8% females (χ2 = 3.2, p = 0.2). There was a positive correlation between pulmonary hypertension and size of VSD and ASD, although the correlation was not significant (Pearson correlation coefficient = 0.01 and 0.4, p = 0.9 and 0.1 respectively). Children with AV canal defect had higher odds of developing most clinical symptoms and pulmonary hypertension than children with other congenital heart disease and this is statistically significant.

**Conclusion:**

Majority of children with AV canal defect presented with pulmonary hypertension. These children present with higher odds of having pulmonary hypertension and clinical symptoms than children with other types of congenital heart disease.

## Introduction

Atrio-ventricular canal (AV canal) defect is a spectrum of congenital heart malformations characterised by a common atrioventricular junction with deficient atrioventricular septation. The incomplete type present with separate atrioventricular valvar orifices despite a common junction, while in the complete AV canal defect, the valve is shared.^1^ The prevalence of AV canal defect varies from 0.24/1000 live births to 0.31/1000 live births.^2^ Children with AV canal defect constitute about 2% of all cardiac malformations.^1^,^3^

Children with AV canal defect presents with various degrees of symptoms such as tachypnoea, recurrent respiratory tract infections, poor feeding, and failure to thrive. These symptoms are usually present by 6–8 weeks due to a fall in pulmonary vascular resistance and blood flow through the large interventricular communication with or without incompetence of the common atrioventricular valve.^1^–^3^ Pulmonary hypertension occurs from excessive pulmonary flow and elevated pulmonary artery pressure via a large venticular septal defect (VSD). Irreversible pulmonary vascular disease could occur by 2 years of age or earlier in infants with Down syndrome.^4^

In children with complete AV canal defect and a small interventricular component, or with ostium primum ASD where atrioventricular valve regurgitation is minimal, cardiac failure is rare and pulmonary hypertension may be minimal or absent in infancy and childhood. Without surgery, however, there is considerable longer-term morbidity and mortality with only 25% survival beyond 40 years of age.^4^ Much has not been done in this locale on the risk of pulmonary hypertension among children with AV canal when compared with children with other congenital heart disease. This work is therefore aimed at determining the risk of pulmonary hypertension and clinical profile in children with AV Canal defect compared with children with other forms of congenital heart disease.

## Methodology

### Study Area

This is a descriptive study, carried out in three institutions including the University of Nigeria Teaching Hospital, Niger Delta University and blessed children hospital all in Enugu, over a -six -year period from March 2015 to March 2020. Three hundred and sixty-seven (367) echo- cardiography reports on children with both congenital and acquired heart disease over a -five-year period was collated. Data on children with AV canal defect was ex- tracted from the records over the same period, and fac- tors such as prevalence, nutritional status, syndromic cor- relates, type of AV canal and clinical presentation were documented. Children who had echocardiographic diag- nostic criteria for either complete or incomplete AV canal defect were enrolled.

### Study Population

Children aged 1 month -18 years who had echocardiography over a five period were recruited in the study. Diagnosis of AV canal defect was made by echocardiography. Cognizance of loss of offsetting, primum Atria-septal defect (ASD), large inlet ventricular septal (VSD), Atria-ventricular valve regurgitation was taken in making diagnosis.

### Study design

A descriptive study that assessed echocardiographic features of children with AV canal defect (these include demographic features, clinical features and presence of pulmonary hypertension) were documented. Children with AV canal defect who fulfilled the inclusion criteria were consecutively recruited into the study.

### Sample Size Estimation

The minimum sample size used in this study was calculated using the formula:

$$\frac{{\text{n = (Z}}_{\alpha }+{\text{Z}}_{\text{1-β}}{)}^{2}\;{\left({\text{p}}_{\text{1}}{\text{ (1-p}}_{\text{1}})+{\text{p}}_{\text{2}}(1-{\text{p}}_{\text{2}})\right)}^{5}}{{\left({\text{p}}_{\text{1}}-{\text{p}}_{\text{2}}\right)}^{2}}$$

This brings the final sample to 43.

### Sample selection of clinical symptoms

Clinical symptoms from children with AV canal defect and those with other cardiac defects were ascertained during the study.

### Estimation of Pulmonary hypertension

Pulmonary hypertension was calculated for children with incomplete AV canal by adding the value of tricuspid regurgitant velocity in m/s to right atrial pressure or central venous pressure (CVP) which is 10mmHg.6 Values above 30mmHg is regarded as pulmonary hypertension. The presence or absence of pulmonary stenosis was also considered when estimating pulmonary hypertension in these children. We used other criteria, as stated by Kushwaha 7 et al, for diagnosis of pulmonary hypertension in children with complete AV canal defect who had common AV valve. This includes the sum of pulmonary regurgitation diastolic gradient and right atrial pressure. Mean PAP≈PR max PG+Rap.

### Statistical analysis

The data was analysed with the IBM SPSS statistics for windows, version 20 (IBM Corp, Chicago). Categorical variables were analysed in form of proportions and percentages and presented in form of tables. Discrete variables including age, weight and height were analysed and summarized as means and standard deviations. Difference in proportions was analysed using Chi-square test and difference in means by Student T-test. Odd ratio was used to assess the risk of association between clinical symptoms and pulmonary hypertension in patients with AV canal defect and other cardiac defects. Probability (p) value was set at p < 0.05.

## Results

There were 367 patients diagnosed of congenital heart diseases over the period of six years and the prevalence of atrioventricular canal defect was 11.7% (43/367). The patients with AV canal defects (subjects) were made of 53.5% males and 46.5% females. The control group (patients with other congenital cardiac abnormalities) included 51.1% males (166/325). There was no significant difference in the sex distribution of subjects and control group (χ2 = 0.20, p = 0.65). The other cardiac defects are as in table 1.

**Table 1:** Frequency of cardiac lesions among the participants

Congenital heart defect	Frequency (n=1162)	Percent (%)
ASD	39	10.6
Truncus arteriosus	3	0.8
DORV	3	0.8
PS	2	0.5
VSD	149	40.6
PDA	70	19.1
TOF	51	13.9
TGA	7	1.9
AV canal defect	42	11.4
Aortic stenosis	1	0.3
Total	367	100

ASD; atrial septal defect, DORV; double outlet right ventricle, PS; pulmonary stenosis, VSD; ventricular septal defect, PDA; patent ductus arteriosus, TGA; transposition of great arteries

The demographic characteristics of the subjects and controls are illustrated in table 2. Among the subjects, 53.5% (23/43) are males while 51.1% (166/325) of the controls are males and gender difference were not significant (χ 2 = 0.2, p = 0.6). The mean age of subjects, 24.7±47.5 months were lower compared with that of the control group, 39.7±48.5 months. However, the difference in mean age was not statistically significant (t = -1.9, p = 0.06).

**Table 2:** Demographic and nutritional status of patients with AV canal defect

Variable	Subjects n (%	Controls n (%)	χ^2^	P value
Sex				
Male	23 (53.5)	166 (51.1)		
Female	20 (46.5)	159 (48.9)	0.2	0.6

Age group				
Infants	30 (70.0)	144 (44.3)		
Preschool	7(16.3)	111(34.2)	11.0	0.01
School age	4 (9.3)	39 (12.0)		
Adolescents	2 (4.6)	31 (9.5)		

+Nutritional status				
Wasted	9 (30.0)	48 (34.4)		
Stunted	8 (26.7)	22 (15.7)	5.2	0.5
Wasted and stunted	6 (20.0)	71 (50.7)		

+ was based on body mass index for age Z-score (BAZ) and height/length Z-score for age (HAZ). 30 of the subjects and 140 of the controls had complete data set for assessing nutritional status

With regards to echocardiographic diagnosis, 86.0% (37/43) had AV canal defect alone while 14.0% had AV canal defect and other associated cardiac defects. The associated defects included patent ductus arteriosus (50.0%) and atrial septal defect, tetralogy of fallot and dextrocardia being 16.7% (1/6) each. Out of the 43 children with AV canal, (6.98%) 3/43 were incomplete while (93.02%) 40/43 were complete AV canal defect. Two point three two (2.32%) 1/43 of the children had a TOF like AV Canal (2.32%) 1/43 had Eisenmenger's syndrome while none had associated isolated pulmonary stenosis.

A large proportion of the patients (77.5%) had pulmonary hypertension. Among the 31 patients with pulmonary hypertension, 45.2% were males compared 54.8% females (χ2 = 3.2, p = 0.2)

The mean pulmonary regurgitation velocity among children with AV canal ranges from 1.08m/s to 4.95m/s with a mean of 3.02± 2.7m/s.

There was positive correlation between pulmonary hypertension and size of VSD and ASD, although the correlation was not significant (Pearson correlation coefficient = 0.01 and 0.4, p = 0.9 and 0.1 respectively). There was no significant difference in pulmonary hypertension values among children with complete or incomplete AV canal defect as in Table 3.

**Table 3:** proportion of those that developed pulmonary hypertension with regards to the type of AV canal defect (Complete versus incomplete)

Type of AV canal defect	Pulmonary hypertension		
	Yes	No	Total
Complete	29	11	40
Incomplete	2	1	3
Total	31	12	43

χ2 = 0.05, p = 0.85

The risk (odd ratio) of a child with AV canal defect developing some clinical features when compared with children with other congenital heart defects is summarized in Table 4. Children with AV canal defect are 96 times more likely to have cyanosis, 35 times more likely to have fast breathing and 29 times more likely to present with cough compared with children with other congenital heart diseases. This also applies to other symptoms as seen in Table 4.

**Table 4:** Odds of developing some clinical symptoms in AV canal defect

Symptom	OR	P value	95% CI
Cough	29.14	<0.0001	6.70 – 126.80
Fast breathing	35.95	0.0008	4.41 – 292.9
Failure to thrive	3.80	0.02	1.20 – 12.17
Cyanosis	96.55	0.002	5.42 – 1721.0
Hypertension	6.76	<0.0001	2.89 – 15.81
Excessive sweating	21.54	0.04	1.09 – 432.2
Easy fatigability	0.88	0.81	0.31 – 2.50
Down syndrome	27.64	<0.0001	10.1 – 75.64

n (number of patients with clinical feature), N (number of patients complete data on clinical feature).

## Discussion

This study has shown a higher prevalence of pulmonary hypertension in children with AV canal defect when compared with other types of congenital cardiac anomaly. Several studies have documented lower prevalence of AV canal in children with congenital heart disease when compared with this present study.^8^–^12^ However, no study had compared these prevalence values of AV canal with that of other congenital heart disease as seen in this study.

Pulmonary hypertension is one of the most dangerous signs in children with AV canal defect with a very high morbidity and mortality. This can lead to increased pulmonary vascular resistance, increase remodelling, heart failure and death. The mechanism of pulmonary hypertension in AV canal defect could be explained by progressive vascular remodelling arising from a left-to-right shunt. If the shunt lesion is large, there will be increase pulmonary blood flow to trigger a pathological mechanism with resultant endothelial dysfunction, hypertrophy of the smooth muscle, with attendant progressive distortion and proliferation of the pulmonary vasculature.^10^,^11^ If the disease progresses, there will be consequential increase in the pulmonary vascular resistance which could be irreversible, in most cases.^10^,^11^

This study showed a very high risk of pulmonary hypertension and higher odds of developing clinical symptoms among children with AV canal defect when compared with children with other forms of congenital heart disease. Though all large, unrestrictive shunt defects may be associated with PAH and elaboration of clinical symptoms; early development of severe clinical symptoms and pulmonary vasculopathy is particularly frequent in complete atrioventricular septal defects.^10^ Nearly 100% of un-operated patients with these anomalies will develop PAH. This is not so with other congenital heart defect.^11^ For instance, un-operated children with large, unrestrictive VSDs or ASDs are also at risk of developing pulmonary hypertension and clinical symptoms.^12^–^17^

This study shows that few children who presented with AV canal and obstructive outflow lesion like pulmonary hypertension did not present with pulmonary hypertension nor did they show any serious risk of worsening clinical symptoms. This could be explained by the protective effects offered by the lungs by the obstructive pulmonary valve.

There was positive correlation between pulmonary hypertension and size of VSD and ASD. This was also corroborated in some studies.^18^–^20^ For instance, it is noted that the risk of developing pulmonary hypertension is not only associated with the size ofthe atrial septal defect (ASD) but also dependent on the compliance of the right ventricle, i.e. magnitude of the left-to-right shunt. The volume load of the pulmonary circulation will be strongly influenced by additional left heart lesions and left ventricular dysfunction.^18^–^21^

Children with AV canal defect had higher odds of developing most clinical symptoms and pulmonary hypertension than children with other congenital heart disease and this is statistically significant. This could also be explained by the fact that post-tricuspid lesions with left-to-right shunts especially AV canal defect are usually at a high-pressure level, which may lead to volume overload on the left ventricle and pulmonary over- circulation. If the post-tricuspid defects are large enough as seen in our study, pulmonary hypertension ensues and this occurs within two years of life compared to other types of post-tricuspid shunt lesion which occur a bit later.^21^,^22^

In fact, if worsening clinical symptoms caused by pulmonary hypertension is not addressed, about 50% of children with AV canal defect will develop supra-systemic PVR with shunt reversal, the so-called Eisenmenger complex. It is documented that children with pulmonary hypertension from AV canal defect have a 5-year mortality that is comparable to those with idiopathic/heritable PAH (29% vs 25%).^22^

## Conclusion

Majority of children with AV canal defect presented with pulmonary hypertension. These children present with higher odds of having pulmonary hypertension and clinical symptoms than children with other types of congenital heart disease.

## Recommendations

Early screening and prompt treatment of pulmonary hypertension in children with AV canal is expedient to avert several morbidities and mortality associated with the defect.

## Strength of the study

The study is bestowed with a large sample size of children with congenital heart disease. In addition, its multicenter nature gives the paper an edge. Moreover, this is the first time this type of work is done in Enugu. It therefore provides a data base for future studies.

## Limitations

A nation-wide analysis of children with AV canal defect would be worthwhile.

## Declarations

Ethics approval and consent to participate: This complies with national guidelines.^23^ All procedures performed in studies involving human participants were in accordance with the ethical standards of the institutional and/or national research committee and with the 1964 Helsinki declaration and its later amendments or comparable ethical standard. Ethical approval was obtained from the Ethics and Research committee of the University of Nigeria Teaching hospital Enugu (IRB number of 00002323).
